# Annual estimates of occupancy for bryophytes, lichens and invertebrates in the UK, 1970–2015

**DOI:** 10.1038/s41597-019-0269-1

**Published:** 2019-11-05

**Authors:** Charlotte L. Outhwaite, Gary D. Powney, Tom A. August, Richard E. Chandler, Stephanie Rorke, Oliver L. Pescott, Martin Harvey, Helen E. Roy, Richard Fox, David B. Roy, Keith Alexander, Stuart Ball, Tristan Bantock, Tony Barber, Björn C. Beckmann, Tony Cook, Jim Flanagan, Adrian Fowles, Peter Hammond, Peter Harvey, David Hepper, Dave Hubble, John Kramer, Paul Lee, Craig MacAdam, Roger Morris, Adrian Norris, Stephen Palmer, Colin W. Plant, Janet Simkin, Alan Stubbs, Peter Sutton, Mark Telfer, Ian Wallace, Nick J. B. Isaac

**Affiliations:** 1 Centre for Ecology & Hydrology, Maclean Building, Benson Lane, Wallingford, Oxfordshire OX10 8BB UK; 2 Centre for Biodiversity and Environment Research, University College London, Gower Street, London, WC1E 6BT UK; 3 RSPB Centre for Conservation Science, RSPB, the Lodge, Sandy, Bedfordshire SG19 2DL UK; 4 Department of Statistical Science, University College London, Gower Street, London, WC1E 6BT UK; 5 British Bryological Society, c/o Biological Records Centre, Centre for Ecology & Hydrology, Wallingford, Oxfordshire OX10 8BB UK; 6 Soldierflies and Allies Recording Scheme, c/o Biological Records Centre, Centre for Ecology & Hydrology, Wallingford, Oxfordshire OX10 8BB UK; 7 UK Ladybird Survey, c/o Biological Records Centre, Centre for Ecology & Hydrology, Wallingford, Oxfordshire OX10 8BB UK; 8 National Moth Recording Scheme, Butterfly Conservation, Manor Yard, East Lulworth, Wareham, Dorset BH20 5QP UK; 9 Soldier Beetles, Jewel Beetles and Glow-worms Recording Scheme, c/o Biological Records Centre, Centre for Ecology & Hydrology, Wallingford, Oxfordshire OX10 8BB UK; 10 Dipterists Forum, Hoverfly Recording Scheme, c/o Biological Records Centre, Centre for Ecology & Hydrology, Wallingford, Oxfordshire OX10 8BB UK; 11 Terrestrial Heteroptera Recording Scheme - Shield bugs and allied species, c/o Biological Records Centre, Centre for Ecology & Hydrology, Wallingford, Oxfordshire OX10 8BB UK; 12 British Myriapod and Isopod Group, Centipede Recording Scheme, c/o Biological Records Centre, Centre for Ecology & Hydrology, Wallingford, Oxfordshire OX10 8BB UK; 13 Grasshoppers and Related Insects Recording Scheme, c/o Biological Records Centre, Centre for Ecology & Hydrology, Benson Lane, Crowmarsh Gifford, Wallingford, OX10 8BB UK; 14 Aquatic Heteroptera Recording Scheme, c/o Biological Records Centre, Centre for Ecology & Hydrology, Wallingford, Oxfordshire OX10 8BB UK; 15 Terrestrial Heteroptera Recording Scheme - Plant bugs and allied species, c/o Biological Records Centre, Centre for Ecology & Hydrology, Wallingford, Oxfordshire OX10 8BB UK; 16 Weevil and Bark Beetle Recording Scheme, c/o Biological Records Centre, Centre for Ecology & Hydrology, Wallingford, Oxfordshire OX10 8BB UK; 17 Staphylinidae Recording Scheme, c/o Biological Records Centre, Centre for Ecology & Hydrology, Wallingford, Oxfordshire OX10 8BB UK; 18 Spider Recording Scheme, British Arachnological Society, c/o Biological Records Centre, Centre for Ecology & Hydrology, Wallingford, Oxfordshire OX10 8BB UK; 19 Dragonfly Conservation Group, British Dragonfly Society, c/o Biological Records Centre, Centre for Ecology & Hydrology, Wallingford, Oxfordshire OX10 8BB UK; 20 Chrysomelidae Recording Scheme, c/o Biological Records Centre, Centre for Ecology & Hydrology, Wallingford, Oxfordshire OX10 8BB UK; 21 Dipterists Forum, Cranefly Recording Scheme, c/o Biological Records Centre, Centre for Ecology & Hydrology, Wallingford, Oxfordshire OX10 8BB UK; 22 British Myriapod and Isopod Group, Millipede Recording Scheme, c/o Biological Records Centre, Centre for Ecology & Hydrology, Wallingford, Oxfordshire OX10 8BB UK; 23 Riverfly Recording Schemes: Ephemeroptera, c/o Buglife Scotland, Balallan House, 24 Allan Park, Stirling, FK8 2QG UK; 24 Riverfly Recording Schemes: Plecoptera, c/o Buglife Scotland, Balallan House, 24 Allan Park, Stirling, FK8 2QG UK; 25 Conchological Society of Great Britain and Ireland, c/o Biological Records Centre, Centre for Ecology & Hydrology, Wallingford, Oxfordshire OX10 8BB UK; 26 Gelechiid Recording Scheme, c/o Biological Records Centre, Centre for Ecology & Hydrology, Wallingford, Oxfordshire OX10 8BB UK; 27 Lacewings and Allies Recording Scheme, 14 West Road, Bishops Stortford, Hertfordshire, CM23 3QP UK; 28 British Lichen Society, c/o School of Natural and Environmental Sciences, Newcastle University, Newcastle upon Tyne, NE1 7RU UK; 29 Ground Beetle Recording Scheme, c/o Biological Records Centre, Centre for Ecology & Hydrology, Wallingford, Oxfordshire OX10 8BB UK; 30 Riverfly Recording Schemes: Trichoptera, c/o Biological Records Centre, Centre for Ecology & Hydrology, Wallingford, Oxfordshire OX10 8BB UK

**Keywords:** Biodiversity, Ecological modelling, Macroecology

## Abstract

Here, we determine annual estimates of occupancy and species trends for 5,293 UK bryophytes, lichens, and invertebrates, providing national scale information on UK biodiversity change for 31 taxonomic groups for the time period 1970 to 2015. The dataset was produced through the application of a Bayesian occupancy modelling framework to species occurrence records supplied by 29 national recording schemes or societies (n = 24,118,549 records). In the UK, annual measures of species status from fine scale data (e.g. 1 × 1 km) had previously been limited to a few taxa for which structured monitoring data are available, mainly birds, butterflies, bats and a subset of moth species. By using an occupancy modelling framework designed for use with relatively low recording intensity data, we have been able to estimate species trends and generate annual estimates of occupancy for taxa where annual trend estimates and status were previously limited or unknown at this scale. These data broaden our knowledge of UK biodiversity and can be used to investigate variation in and drivers of biodiversity change.

## Abstract (summary)

**Table:** 

Measurement(s)	Occupancy • Species • biodiversity assessment objective
Technology Type(s)	occupancy modelling • biological records • Trends
Factor Type(s)	species
Sample Characteristic - Organism	Lichens • Invertebrates • Bryophytes
Sample Characteristic - Location	United Kingdom

Machine-accessible metadata file describing the reported data: 10.6084/m9.figshare.9977426

## Background & Summary

Knowledge on the status and trends of biodiversity is essential for the conservation of threatened species and for the monitoring of progress towards biodiversity targets^1^. To date, UK scale analysis of annual biodiversity status has been restricted to well-studied taxa such as birds^2^, butterflies^3^, bats^4^, Odonata^5^, some moths^6^ and a subset of “priority” species^7^. As a result, there are many taxa for which only coarse-scale measures of change are available with most invertebrate groups being a major gap due to a lack of abundance data. However, species occurrence records are fine-grained data available for many taxa and offer an alternative data source that can be used for estimating annual measures of biodiversity change.

Occurrence records are presence-only data documenting observations of species at known dates and locations. Within the UK, vast amounts of such occurrence data, known as biological records, are collected by volunteers and collated by recording schemes and societies and have been used extensively to produce species atlases and assess species range shifts^8^. These data offer greater taxonomic breadth than structured abundance data and have rarely been used to detect long-term change^9–13^. This limited use is due partly to the unstructured collection process which results in at least four forms of bias: the uneven detectability of species across space and time, uneven sampling effort per visit, uneven spatial coverage and uneven recording intensity over time^14,15^. These biases present challenges when estimating temporal change, however, methodological techniques have been developed that attempt to account for some forms of bias. One technique that has been increasingly used for the analysis of occurrence records is occupancy modelling^7,16–19^.

Occupancy models incorporate the data collection process to account for imperfect detection^17,20,21^. When compared with other methods developed for the estimation of trends from occurrence records, occupancy models have been shown to be the most capable of addressing associated biases, if the detection process is appropriately specified^21^. However, their use has been limited to taxa that have a high recording intensity including birds^22^, dragonflies^5,19^ and butterflies^17,23^. Outhwaite *et al*. extended a previous Bayesian occupancy modelling framework, to increase the precision of occupancy estimates via the use of a random walk prior on the year effect of the state model^24^. This formulation allows information to be shared between years in a natural way and facilitates the application of such models to datasets of a low recording intensity that were not previously considered for practical use. Therefore, through the application of a modelling framework based on that of Outhwaite *et al*.^24^ we have produced a 45-year dataset of annual occupancy estimates for 5,293 UK bryophyte, lichen and invertebrate species.

This dataset presents a long-term measure of change in species occupancy at a national (UK and GB) and nation-specific scale (England, Scotland, Wales and Northern Ireland) using fine grained (1 × 1km) data. This represents new information for the taxa covered by this study. By providing the outputs of this analysis we hope to promote research into UK biodiversity change, particularly for those taxonomic groups that have received less attention in the context of national-scale trends at fine scales. It is hoped that these data will provide the basis of future aggregate measures of UK biodiversity change and enable the investigation of drivers of change.

## Methods

**Fig. 1:**
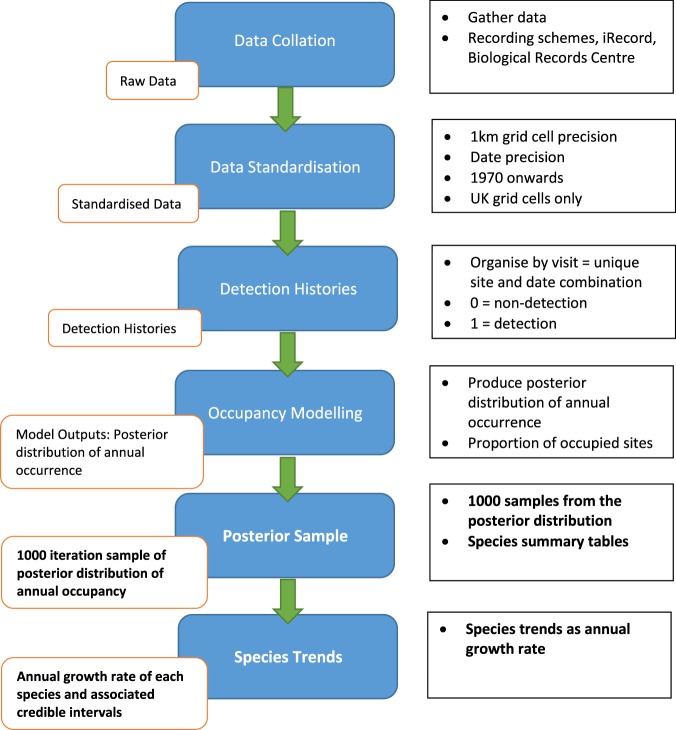
The workflow to produce the datasets presented in this study. Major steps are shown in the blue boxes with the data type generated within the orange boxes. Associated details are given next to each step. Datasets provided alongside this paper are shown in bold.

The raw data underpinning these models were occurrence records collated from 29 UK or Great Britain (GB) based recording schemes and societies, with additional data from the Biological Records Centre, Wallingford, and the iRecord database (https://www.brc.ac.uk/irecord/). These data were standardised to ensure all datasets met the required criteria (see methods) and had undergone review by experts of each species group. The standardised data were then organised into detection histories to enable their use within an occupancy modelling framework. Our Bayesian occupancy model was fitted for each species and provided annual estimates of occupancy for each country analysed. From these estimates, growth rates of species occupancy were calculated. The full workflow is described in Fig. 1 with the following sections describing each part of the workflow. The outputs from this study include 1000 samples from the posterior distribution of the annual occupancy estimates for each country analysed, large-scale trend estimates for each species in the form of annual growth rates as well as additional metadata.

### Data collation

Data were collated from recording schemes and societies that support recording networks and collect and verify occurrence records on UK species. Twenty-nine schemes granted the use of their data for this analysis. For some taxonomic groups, mainly where the number of records provided by the scheme was low, further data were acquired from the databases of the Biological Records Centre and from the iRecord system. iRecord is a website and associated mobile phone application that has been designed to support recording schemes and societies in the collation, management, quality assurance and sharing of wildlife observations (https://www.brc.ac.uk/irecord/). Only records that had been marked as “accepted” by an iRecord verifier were used^25^. These sources can be considered different routes for accessing the same form of data. Some of the raw data are available through the NBN Atlas https://nbnatlas.org/, Online-only Table 1), although in some cases the publicly-available data is at a coarse spatial resolution or limited in time compared to that used here. For some schemes, the data had to be requested and supplied directly by the BRC or the scheme. Users desiring access to these data should first check the availability via the NBN Atlas and/or contact the scheme directly (contact information can be found on the relevant BRC scheme pages (https://www.brc.ac.uk/recording-schemes).

The occurrence records used in this analysis are presence-only data of a species and consist of a what, when and where: what species was observed, when it was observed and where it was observed. In most cases, one scheme is associated with one taxonomic group. In the case of data from the Bees, Wasps and Ants Recording Society, the dataset was split into three separate datasets for analysis, one for each taxonomic group, as these taxa are not considered to be recorded as a complete entity by all members of the society. The data collation step resulted in the generation of 31 “raw” datasets, one for each taxonomic group assessed.

### Data standardisation

The resolution of record location and date vary in these data, particularly for data from earlier decades. Record location is represented by a British or Irish grid reference, but the resolution differed between records (e.g. 10 m, 100 m, 1 km, 2 km, 10 km). The date format of a record also varied or was unknown. The most precise records state on which day a species was recorded, but some older records state a year or range of years. These differences mean that the datasets needed to be standardised to ensure a collection of records with the same level of spatial and temporal precision. For our model, records are required with day level precision to maximise the number of replicates within a year from independent visits. Replicate visits within a closure period (here one year) are essential for estimating species detectability within the occupancy model^26^. Records where the date of the record was unknown were removed from the dataset. A 1 × 1km grid cell precision for location was chosen as this would provide the greatest number of spatial replicates across the time period of interest for most taxa. Any records with a more precise location were scaled up to 1 km resolution. Any records with a less precise location were removed from the dataset. Only records from 1970 onwards were retained within the datasets as, in general, the number of records at a 1 × 1km precision before 1970 was low for most taxa. A check was also carried out to ensure that only records from grid cells within the UK (England, Northern Ireland, Scotland and Wales) were retained, excluding data from the Channel Islands, the Republic of Ireland and the Isle of Man.

The supplied species names for each taxonomic group were also checked. Any records that were made to a taxonomic level higher than species were excluded from the dataset (but see below regarding species aggregates). Scheme organisers were contacted to aid in the checking of the species lists to ensure that synonyms representing the same species were not used in isolation. Spelling mistakes were also identified and corrected where necessary. In specific cases, certain species were modelled as aggregates of species. This was carried out upon advice from the scheme organisers and was due to changes in taxonomy during the period of interest resulting in records where species identity could not be certain, difficulties in identification of species by recorders, or differences in what people actually record under a specific name. After these checks were carried out, any duplicate records were then removed from the dataset.

The standardisation process resulted in 31 datasets covering 10,750 species and 24,118,549 individual species records (Online-only Table 1). Note that the coverage of countries within the UK varies between schemes. Some groups, therefore, were only analysed at the scale of Great Britain (GB, includes England, Scotland and Wales) rather than at the UK scale (includes England, Scotland, Wales and Northern Ireland). The spatial coverage of the records within each of these standardised datasets can be seen in the maps provided in Supplementary Fig. S1.

### Organisation of detection histories

The standardised data were organised into detection histories as first applied to presence-only data by Kéry *et al*.^16^. In this format the data were reorganised into visits (unique combinations of 1 × 1km grid cell and date), with 0 s or 1 s assigned to denote whether a species was detected (1) or not detected (0) during each visit. This step enables the use of presence-only data within a framework that requires information on non-detection. Detections were extracted directly from the data as a record of a species at a known date and location. There are two types of non-detection: true absences and false absences: where the species has been overlooked. Non-detections were inferred from the detection of other species within that taxonomic group when the focal species was not observed. For example, if ant species A was not detected during a visit, but ant species B, C and D were detected then this would be classified as a non-detection of ant species A. We assume that species A was available phenologically to be detected at the same time as other species. It is possible to include phenological information into the detection submodel^27^: our preliminary investigations showed that including phenology made little difference to the resulting estimates but dramatically slowed the convergence time, so we report results without terms for phenology. Our long-term trend estimates therefore assume that the distribution of recording effort throughout the year has remained approximately constant over time. List length is the number of species recorded during a visit, this parameter is used in the occupancy modelling framework as a proxy for sampling effort^28^ (see later section). The detection history dataset and the list length of each visit were fed into the occupancy modelling framework for the analysis.

The organisation of the standardised data into detection histories was carried out using the function “*formatOccData”* in the R package *sparta*^29^. *sparta* is an R package that contains various methods for the analysis of unstructured occurrence records and is freely available on GitHub (https://github.com/BiologicalRecordsCentre/sparta). An example of how to use this function to generate model-ready datasets can be found in Supplementary File 1.

### The occupancy model

The occupancy model used here is based on the “random walk” model of Outhwaite *et al*.^24^. The name refers to the use of a random walk prior on the year effect of the state model, which was found to improve precision in occurrence estimates, particularly for datasets with a low recording intensity (most of the input datasets in this study). This development has enabled the much broader application of occupancy modelling than was previously possible.

The model is a hierarchical model split into two distinct sub models: the state model and the observation model. The state model describes the true occupancy state, *z*_*it*_, of site *i* in year *t* and is defined by Eqs (1) and (2). *z*_*it*_ will be 1 when a site is occupied and 0 if the site is not occupied. *z*_*it*_ takes a Bernoulli distribution:

1 $${z}_{it} \sim {\rm{Bernoulli}}\left({\psi }_{it}\right).$$

where the logit of the probability of occurrence, *ψ*_*it*_, varies with year and site:

2 $${\rm{logit}}\left({\psi }_{it}\right)={\rm{\log }}\left(\frac{{\psi }_{it}}{1-{\psi }_{it}}\right)={b}_{t}+{u}_{i},$$

*b*_*t*_ and *u*_*i*_ denote year and site effects respectively.

For the model used here, the state model year effect was split into four regions to allow the estimation of occupancy for each country within the UK, as well as the aggregate occupancy at UK and/or GB level. This means that, instead of having a single year effect in the state model as shown in Eq. 2, there is a year effect associated with each country, hereafter termed region. Specifically, let *r*(*i*) be the region (England, Northern Ireland, Scotland or Wales) in which site *i* is located, then:

3 $${\rm{logit}}\left({\psi }_{it}\right)={\rm{\log }}\left(\frac{{\psi }_{it}}{1-{\psi }_{it}}\right)={b}_{tr(i)}+{u}_{i},$$

where ${b}_{tr(i)}$ is the year effect for year *t* in region *r* in which site *i* is found.

The observation sub model describes the data collection process and is conditional on the true occupancy state *z*_*it*_. *p*_*itv*_ represents the probability that a species will be observed on a single visit, given the species is present at that site. The observation, *y*_*itv*_, is then described as being drawn from a Bernoulli distribution conditional on the true occupancy state:

4 $${y}_{itv}|{z}_{it}\sim {\rm{B}}{\rm{e}}{\rm{r}}{\rm{n}}{\rm{o}}{\rm{u}}{\rm{l}}{\rm{l}}{\rm{i}}({p}_{itv}.{z}_{it})$$

This means that a species can only be detected at a given site if it is truly present. We therefore assume that there are no false positive observations (for example incorrect species identifications) within the dataset. Given that our occurrence records are curated and verified by recording schemes and their organisers, this is likely to be a reasonable assumption. However, our long-term trend estimates will be biased if there is a directional trend in the rate of misidentification. A model extension has been developed that can deal with false positives^30^, but it has a small effect on overall occupancy.

Variation in detection probabilities *p*_*itv*_, per visit are described as follows by Outhwaite *et al*.^24^:

5 $${\rm{logit}}\left({p}_{itv}\right)={\rm{\log }}\left(\frac{{p}_{itv}}{1-{p}_{itv}}\right)={a}_{t}+c\,{\rm{\log }}\,{L}_{itv},$$

where *a*_*t*_ is a year effect and *L*_*itv*_ is the list length, that is the number of species recorded during a single visit. In this form, *c* represents the change in the detectability of the focal species as the list length increases. In using this formulation, the assumption is that there is likely to be a positive relationship between the number of species recorded on a visit and the probability of a species being detected. The suggestion being that more time was spent looking and so greater sampling effort expended. However, Eq. (5) imposes a specific mathematical form on the relationship between list length and species detectability, and this form may not be justified for all the species considered here. This continuous option is also likely to result in higher assumed detection in the south due to a general higher species richness than occurs in the north. Therefore, rather than using a continuous specification of list length we have chosen to use a categorical specification in which detectability is classified according to whether a species is recorded on a list of length 1, 2–3 or 4 + records. This alternative classification of list length was considered by Van Strien *et al*.^17^ as a more flexible alternative to the continuous specification where detectability does not follow an increase with list length. It also does not assume that each list was a complete list of species recorded during that visit. As we were looking to apply this method across many thousands of species, a single option applied broadly to all groups was used, although we recognise that this may be less suitable for the few high richness groups considered in this study. In the model implemented here, Eq. (5) is replaced with the following:

6 $${\rm{logit}}\left({p}_{itv}\right)={\rm{\log }}\left(\frac{{p}_{itv}}{1-{p}_{itv}}\right)={a}_{t}+{\beta }_{1}\ast datatype{2}_{itv}+{\beta }_{2}\ast datatype{3}_{itv},$$

where *β*_1_ and *β*_2_ estimate differences in logit(*p*_*itv*_) for a list length of 2–3 (datatype2) and of 4+ (datatype 3) respectively, relative to a list length of one.

This model is run in a Bayesian framework which requires unknown parameters to be assigned a prior distribution. The prior distribution describes our knowledge of the system before the data were collected. In the model formulation of Outhwaite *et al*.^24^ vague, uninformative priors are set on all parameters except for the year effect of the state model. The preferred prior on this parameter uses a random walk to describe the change in occurrence as similar to that of the previous year with some variation. Here, we apply this to the year effect for year *t* in region *r*, *b*_*tr*_:

7 $${b}_{tr}\sim \{\begin{array}{c}{\rm{N}}{\rm{o}}{\rm{r}}{\rm{m}}{\rm{a}}{\rm{l}}({\mu }_{br},{10}^{4})\,{\rm{f}}{\rm{o}}{\rm{r}}\,{t}=1\\ {\rm{N}}{\rm{o}}{\rm{r}}{\rm{m}}{\rm{a}}{\rm{l}}({b}_{t-1r},{\sigma }_{br}^{2})\,{\rm{f}}{\rm{o}}{\rm{r}}\,{t} > 1\end{array}$$

8 $${\rm{where}},\,{{\rm{\mu }}}_{br} \sim {\rm{Normal}}\left(0,100\right){\rm{,}}$$

9 $${\rm{and}}\,{\sigma }_{br} \sim | {\rm{Student}} \mbox{-} t\,{\rm{on}}\,{\rm{1}}\,{\rm{degree}}\,{\rm{of}}\,{\rm{freedom| }}$$

See Outhwaite *et al*.^24^ for further details on the random walk prior.

Priors on all other parameters are set out as in the original paper including the use of the recommended half-Cauchy hyperpriors. These are set as shown in Eq. 9 as the modulus of a Student’s t-distribution on 1 degree of freedom. Information on the setting of initial values can be found in the original paper where the procedure outlined was followed.

The models were fitted using the function “*occDetFunc*” from the R package *sparta*, selecting the random walk model with half-Cauchy hyperpriors and using the categorical specification of list length^29^. Parameters set for the model running process included *nyr* = 2, this means that any sites with fewer than two years of data are dropped from the dataset. Models were fitted to data for the period 1970 to 2015.

The *sparta* package uses a Markov Chain Monte Carlo (MCMC) algorithm to fit the models, using JAGS^31^ via the function *occDetFunc*. This process can be computationally expensive, particularly when datasets consist of a large number of records and/or a large number of species. For small to medium datasets, models were fitted using a computer cluster hosted at CEH, Wallingford. Using this process, species were run in parallel across multiple cores. Large datasets, including the moths, dragonflies, bryophytes and lichens were run on the much larger NERC JASMIN supercomputer. For groups run on the CEH cluster, the MCMC algorithm was run for 40,000 iterations per species with a burn in of 20,000 and a thinning rate of three. This was sufficient to obtain convergence for most of the parameters of interest for most species. Convergence was assessed using the Rhat value^32^, where a value below 1.1 is considered sufficient^33^. For those groups run on JASMIN, the greater size of the datasets meant that these groups took longer to run than those run on the CEH cluster. These groups were run for 20,000 iterations in total with a burn-in of 10,000 and a thinning rate of three. As these groups generally had more data per species, convergence was reached in fewer iterations, so this was considered an acceptable compromise to reduce the overall run time. For a general idea of run time, small data sets with few species take just a few hours when run in parallel on these systems, but large datasets with many species took several weeks. An example of how to run an occupancy model using the *occDetFunc* function in the *sparta* package can be found in Supplementary File 1.

We fitted the model described above to all 10,750 species within the standardised datasets, regardless of the number of records that were available for that species. Species occupancy each year was calculated as a derived parameter within the model as the proportion of occupied sites. This was calculated for each region covered by the model, therefore, estimates are available for each species for multiple regions depending on input data coverage (see Online-only Table 1). A posterior distribution of estimates for each year for each region was therefore generated by the MCMC process.

### Assessing species outputs

As a combination of species rarity, the data standardisation process, and the implementation of the *nyr* parameter (see section on the occupancy model), the number of records available for each species varied considerably. As a result, model outputs for some species were based on very few records. As a model output based on just a few records cannot be considered to contain any valuable information, there was a requirement to set a threshold number of records that a species must have to be considered a part of the dataset described here. After model fitting, we therefore set a threshold of 50 records across the 45 year time period, increasing this threshold made very little difference to multispecies assessments (not presented here) so this value is maintained (see also Outhwaite *et al*.^24^ for examples of species models that achieve useable results based on 50–500 records). Users can increase this threshold for their own use should that be considered appropriate: the number of records contributing to each species output has been provided alongside the data in the repository. Species were also removed if they contain a gap in the dataset where more than 10 consecutive years were lacking records, this was to prevent possible cases where the prior takes over during periods of no data (see supplementary of the original paper describing the random walk model^24^). This reduced the number of species from the 10,750 that models were fitted for to 5,293 that we consider to contain valuable information on species status. Considering that the number of records that contributed to the estimation of the occupancy and trend values, as well as the uncertainty around these estimates, is important, this information has also been provided within the data repository.

### The posterior distribution

The output produced from the occupancy model is a posterior distribution of the occupancy parameter estimated as the proportion of occupied sites for each year, within each region, for each species. These estimates cover the years 1970 to 2015. Some input datasets ended prior to 2015 so estimates are produced for years where data are not available, although the uncertainty (which is quantified via the provided samples from the posterior distribution) will be greater during these years. To make the analysis of these outputs manageable to users, we supply 1000 samples from the occupancy posterior distribution for each region as a part of this dataset.

### Species trends

Long-term species trends were estimated as the percentage annual growth rate of occupancy using the following formula:

10 $$annual\,growth\,rate=\left({\left(\frac{f}{s}\right)}^{\frac{1}{y}}-1\right)\times 100,$$

where, *f* was the occupancy in the final year, *s* was the occupancy in the starting year and *y* was the number of years. The growth rate was calculated for each of the 1000 samples which were then summarised using the mean and 95% quantiles. To avoid extrapolating beyond the scope of the data, the start and end years that were used to calculate the annual growth rate differed between species depending on which years had species observations contributing to the occupancy outputs. For example, if the input dataset for a species only had records of that species between 1974 and 2013, then the posteriors for these years were used as the final and starting years in the formula above. This was considered appropriate as when there are no data at the start of the time period, the prior of the model can have an influence on the result (see supplementary information by Outhwaite *et al*.^24^ for further information). The start and end years per species are detailed alongside the trend estimates in the repository. The precision of the trend estimate is also supplied and is estimated as 1/variance of the 1000 sample trends.

## Data Records

All outputs as a part of this dataset are freely available through the Natural Environment Research Council (NERC) Environmental Information Data Centre (EIDC) within the dataset entitled “Annual estimates of occupancy for bryophytes, lichens and invertebrates in the UK (1970–2015)^34^” and is freely available to download (10.5285/0ec7e549-57d4-4e2d-b2d3-2199e1578d84).

Data presented within this dataset are in three forms:

1. **1000 samples of the posterior distribution** of the proportion of occupied sites for each region per species per year (one file per species).
2. Tables summarising the **mean occupancy per region and associated uncertainty** for each species (one file per species).
3. **Large-scale long-term species trends** derived from the posterior samples as the percentage annual growth rate (one file, row per species).

These data are accompanied by information on the input datasets used to generate these estimates (one file) and information on the origins and changes to species names (one file). All data files are provided in a .csv format.

### Samples of the posterior distribution of species occupancy

The output produced from the model is a posterior distribution of the occupancy parameter estimated as the proportion of occupied sites for each year, for each region, for each species. These estimates cover the years 1970 to 2015 and encompass four regions for GB scale groups (GB, England, Scotland and Wales) and six regions for UK scale groups (UK, GB, England, Scotland, Wales and Northern Ireland). To make the analysis of these outputs manageable to users, we supply 1000 samples from the occupancy posterior distribution for each region. 1000 samples from the posterior were randomly selected for each species:year combination and across each region, these are supplied as a csv file for each species with a row per iteration within each region and a column per year (Table 1). These can be found in the “POSTERIOR_SAMPLES” folder in the repository^34^. Values presented in the year columns of these tables represent the proportion of sites occupied by that species in that region and can be any value between zero and one.

**Table 1:** Example table showing the layout of the samples from the posterior distribution for a species. There is a row per iteration per region and a column per year. Additional columns detail the region, iteration, the species name and the taxonomic group that species belongs to. ‘…’ represents intervening years and regions not shown here.

Group	Species	Region	Iteration	1970	1971	1972	…	…	2015
Ants	Formica aquilonia	GB	1	0.01804	0.02296	0.02132			0.01886
Ants	Formica aquilonia	GB	2	0.00164	0.00205	0.00082			0.01599
Ants	Formica aquilonia	GB	3	0.02747	0.02706	0.02952			0.02501
		…							
		…							

### Model output summary tables

Alongside the samples from the posterior distribution, we also supply the summary table from the model output for each species. This table includes the mean estimate for each year and the associated 95% credible intervals estimated from the complete posterior distribution (Table 2). It also includes the standard deviation and Rhat values for each estimate. The Rhat parameter estimates the convergence of the MCMC chains, a value of 1.1 is usually considered acceptable^33^. A summary table for each species is supplied as a csv file in the “SUMMARY_TABLES” folder of the repository^34^. The numeric values in this table have been rounded to three decimal places.

**Table 2:** An example summary table showing the layout and parameters included. There is a row per year per region. Information columns detail the taxonomic grouping, species name, region and year of the estimates. The remaining columns detail the statistics for that estimate including mean occupancy, 95% credible intervals, the standard deviation and the rhat statistic. ‘…’ represents intervening years not shown here.

Group	Species	Region	Year	Mean	Lower_CI	Upper_CI	Standard_deviation	Rhat
Ants	Formica aquilonia	GB	1970	0.034	0	0.081	0.03	2.937
Ants	Formica aquilonia	GB	1971	0.034	0	0.08	0.03	3.015
Ants	Formica aquilonia	GB	1972	0.034	0	0.08	0.03	3.101
			…					
Ants	Formica aquilonia	GB	2015	0.039	0.006	0.082	0.029	2.833

### Species trends

Species trends, calculated as the percentage annual growth rate are supplied alongside the associated credible intervals, the first and last years used to calculate these trends for each species, the number of years across which the trend estimate is calculated and the number of records of the species (Table 3). The precision of the estimate is also presented. These values are present in a single table in the “Species_Trends.csv” file within the repository^34^. The numeric values in this file have been rounded to three decimal places.

**Table 3:** Table showing the layout of the species trends csv file. Associated information is provided including taxonomic grouping, species name, the number of years of data, the first and last years used to estimate growth rate, and the number of records of this species contributing to the occupancy estimates. The growth rate and 95% credible intervals are then supplied along with the precision of the estimate.

Group	Species	N_Years	First_Year	Last_year	N_Records	Mean_growth_rate	Lower_CI	Upper_CI	Precision
Ants	Formica aquilonia	34	1981	2014	94	−1.038	−4.160	1.861	0.448
Ants	Formica cunicilaria	46	1970	2015	785	−2.059	−4.033	−0.221	1.101

#### Accompanying metadata

Another csv file details information on the input datasets used to generate the results shared (Table 4). This includes the number of records in each input dataset, the name of the recording scheme that provided data, the number of species covered by the input datasets and the number that are covered by the outputs supplied. This table also details the number of visits that meet each list length category specified within the model. This information is taken after the datasets have been standardised and has been supplied in the “Dataset_Information.csv” file within the repository^34^. This file contains all information in Online-only Table 1.

**Table 4:** Example rows of the Dataset_information.csv table.

Group	Country coverage	First_Year	Last_Year	Scheme_Name	N_Records	N_Species	N_Species Outputs	N_Sites	N_Visits	LL_1	LL_2–3	LL4+
Ants	GB	1970	2015	Bees, Wasps and Ants Recording Society	34790	60	29	8988	19530	11749	5906	1875
Aquatic Bugs	GB	1970	2015	Aquatic Heteroptera Recording Scheme	61264	92	51	10254	21014	8701	6697	5616
Bees												

Information on the origin of the species names used is detailed in the “Species_Names.csv” file, also found within the repository^34^. This includes information on why a model was not fitted for a species, advice on aggregations from schemes and any other changes made to species names (Table 5).

**Table 5:** Example of the information provided in the “Species_Names” csv file. This table includes information on all 10,750 species included in the study, the origin of a species name and any information detailing name changes or species aggregations where available.

Group	Species	Name_origin	In_final_dataset	Reason_not_included	Details
Ants	Formica aquilonia	Direct from scheme dataset	Yes	NA	
Ants	Formica cunicularia	Direct from scheme dataset	Yes	NA	
Ants	Formica exsecta	Direct from scheme dataset	No	Didn’t meet criteria	
Ants	Formica fusca	Direct from scheme dataset	Yes	NA	
Ants	Formica lemani	Direct from scheme dataset	Yes	NA	

## Technical Validation

The model used here is based on the “random walk model” tested by Outhwaite *et al*.^24^. The authors tested this model, and other variants, on both simulated data and real world occurrence records (the kind used to produce this dataset). They showed that the random walk model improved the precision of the occupancy estimates and had low bias when estimating known species trends from simulated data. This model is, therefore, arguably the most appropriate for use in this study, particularly due to its improved application to datasets of a low-recording intensity which several the input datasets included here suffer from.

The input datasets were checked and standardised as described in the methods section. Species names within each taxon group dataset were checked by scheme organisers or were compared to online checklists to ensure no synonyms were present alongside preferred species names. Note that all the schemes providing data to these analyses maintain taxon registers integrated into their databases to ensure the taxonomic coherence of all data held, and to ensure conformity with the currently accepted taxonomic standard of that scheme. Scheme organisers also recommended the removal or aggregation of species where it was not certain which species the records were referring to (details in Species_Names.csv file^34^). This could occur, for example, when a single species is split into two separate species. Those species may then be aggregated under one species name if records before the split cannot be identified as one of the two split species. Aggregate species can be identified by *agg*. within the species name. Any species where record identity was questioned, for example because species are very difficult to identify with confidence, were highlighted. These were retained within the dataset to fully inform list length, but models were not fitted for these species. These checks ensure that all data relevant to a taxon can be extracted from scheme databases, even if occurrence records were originally collected against synonyms or at a lower (infraspecific) rank than that of the species. It should be noted that, across the different taxon-focused schemes, decisions regarding the suitability of particular types of species occurrence data for modelling will vary, and these decisions are captured within the dataset metadata where provided (details in Species_Names.csv file^34^). As an example, the Bees, Wasps and Ants Recording Scheme did not consider data relating to species within the *Lasius niger* aggregate as suitable for modelling: taxonomic changes have meant that data collected at different time points under the name “*Lasius niger*” cover two distinct species, and the scheme did not consider a trend at the aggregate level of these concepts to be ecologically meaningful. To give a contrasting example, the British Bryological Society were happy for a trend to be produced for the moss taxonomic concept *Ulota crispa sensu lato (s*.*l*.*)*, an aggregate covering the species *Ulota crispa sensu*^35^ and *Ulota bruchii* (see ref.^36^ for a similar analysis using this aggregate), because this was felt to be both ecologically meaningful, and to make the best use of historic data. Ultimately, species trends produced using species occurrence data must deal with the trade-off between the taxonomic uncertainty attached to any given record and producing the most meaningful assessment of change given the available data. The expert opinion of those who collect and curate such data is an essential accompaniment to automated checks. For reference, species names were also checked using the *taxize* R package^37,38^. The scores generated using the *gnr_resolve* function to check names against the GBIF Backbone Taxonomy register can be found in Supplementary File 2.

The number of records that make up the input datasets for each taxonomic group differed substantially, see “Dataset_Information.csv”^34^ or Online-only Table 1. This will impact the spatial coverage and the number of sites that estimates are based on (maps of the spatial coverage of the standardised input datasets are available in Supplementary Fig. S1).

Convergence of the model parameters of occupancy was assessed using the Rhat statistic. This measure is commonly used to assess the convergence of a model parameter with values less than 1.1 generally considered to be adequate. Due to the size of the datasets and the time taken to run all the models it was not possible to run all models to complete convergence. Set numbers of iterations were therefore undertaken according to the size of the dataset: 40,000 for smaller datasets and 20,000 for larger datasets. We have supplied a summary table for each species that details the mean occupancy values, the standard deviation of the estimates, 95% quantiles of occupancy and the Rhat value so users can check convergence of estimates as well as the uncertainty associated with the mean occupancy estimates, these can be found in the species specific csv files in the “SUMMARY_TABLES” folder of the repository^34^.

The number of records per species within each dataset also varied considerably. In some cases, data standardisation and the removal of sites visited in only a single year (using the *nyr* model parameter) meant that some species were left with very few records. A column detailing the number of records per species after filtering has been supplied to ensure users are aware of the number of records contributing to species estimates. These values can be found in the N_records column of the “Species_Trends.csv” file^34^.

**Fig. 2:**
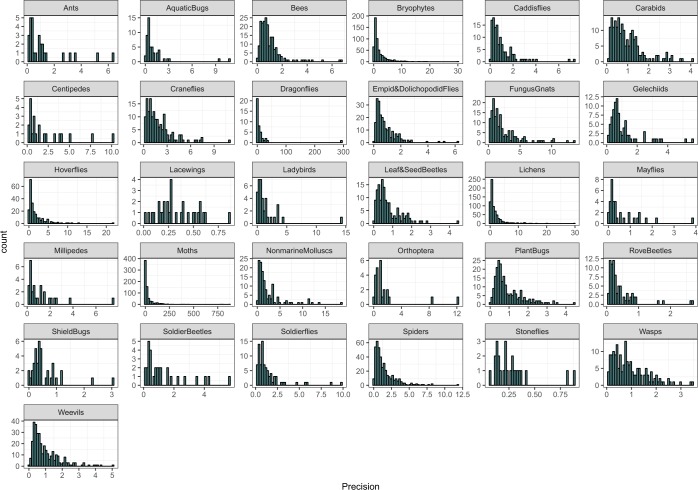
Histogram of the precision estimates of the estimated species trends (annual percentage growth rates). Note that the axes vary across groups.

It was not possible to validate these estimates against an independent source of distribution or occupancy trends, since this is the first time that such information has been produced. As a form of statistical validation, we explore the precision of the trend estimates. Precision of the trend estimates are presented within the Species_Trends.csv file^34^ but are highly variable (Fig. 2) reflecting variance in the number of records available for each species^39^.

## Usage Notes

**Fig. 3:**
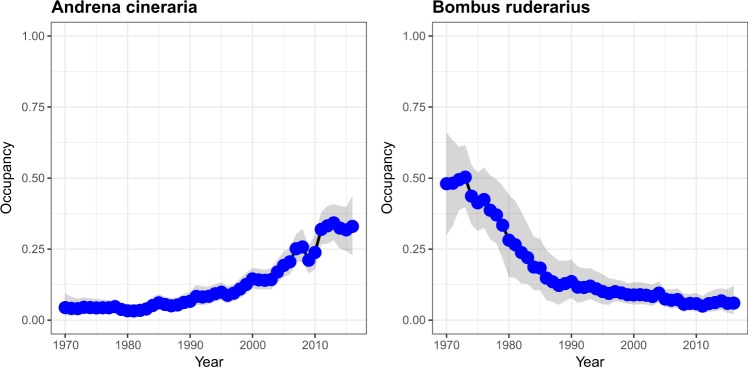
Example plots of species occupancy estimates over time and associated 95% credible intervals for two bee species.

This dataset can be used to assess change in occupancy of single species or an aggregation of species. Plotting the mean estimate from the summary table alongside the associated credible intervals for a species will give you a plot of the occupancy estimates for that species over time (Fig. 3).

When using the outputs provided within this dataset, users need to consider the uncertainty assessments supplied alongside the data. Those species where the uncertainty assessments we consider unreliable (those with fewer than 50 records and gaps of 10 years between records) have been removed from this dataset. However, users are urged to make their own judgement on whether the uncertainties are small enough to provide useful information in the context in which they are being used. Uncertainty can be established by summarising 95% quantiles of the posterior samples or from the supplied 95% credible intervals in the species occupancy summary tables and species trends table. Data users should also make sure to take a note of convergence of parameters contributing to the estimates when using these outputs.

The fitting of the models and analysis of the outputs produced can be carried out using two R packages that have been developed for this purpose. *sparta* is an R package that has been developed to carry out methods for the estimation of species trends from occurrence records^29^. This package is freely available on GitHub: https://github.com/BiologicalRecordsCentre/sparta.

The posterior samples for species can be used to generate aggregate indicators of change in occupancy over time with associated measures of uncertainty for groups of species or for specific regions. Using the posterior samples means that uncertainties can be propagated throughout the analysis. Another R package, *BRCindicators*, has been developed to estimate species trends and generate indicators of change over time from the outputs produced from *sparta* or similar methods. This package is also available on GitHub: https://github.com/BiologicalRecordsCentre/BRCindicators.

## Supplementary information

Supplementary File 1.

Supplementary Figure S1.

Supplementary File 2.

## Data Availability

Code used for taxa specific input data standardisation is not presented. Species name checks and changes were taxa specific and required a lot of manual processing after consultation with scheme organisers. Information on species aggregations, removals and name changes are, however, detailed in the “Species_Names.csv” spreadsheet. Functions for organising data into detection histories and for fitting the specified occupancy model are available in the R package *sparta*^29^. The function *formatOccData* was used to arrange the data into detection histories and to calculate the list length of visits. The function *occDetFunc* was used to run the models. Note that in order to run these models using *sparta*, JAGS must be downloaded separately in order to carry out the MCMC sampling^31^. An example workflow detailing function and model specifications has been supplied within Supplementary File 1. This PDF document runs through each subsection of the methods, except the raw data processing, providing the code used and examples of the outputs produced as a result. Raw data processing was not included since processes were group specific and raw data could not be supplied alongside the outputs due to data provider restrictions.

## Online-only Table

**Online-only Table 1:** Information on the standardised datasets used as inputs into the occupancy modelling framework. The name of the schemes contributing data for each taxon are given as well as information on the data sources, coverage and range of the datasets. The number of species covered by the standardised datasets is given (N species, input), as well as the number of species that results are supplied for as a part of the dataset associated with this paper (N species, outputs). * Denotes those schemes that share some or all of their data via the NBN Atlas either directly from the scheme or via the Biological Records Centre.

Taxonomic Group	Recording Scheme	Additional data sources	Country coverage	Year range of input data	N species, input	Number of records	N species, outputs
Ants	Bees, Wasps and Ants Recording Society	Scheme data only	GB	1970–2015	60	34,790	29
Aquatic Bugs	Aquatic Heteroptera Recording Scheme *	Scheme, BRC and iRecord data	GB	1970–2015	92	61,264	51
Bees	Bees, Wasps and Ants Recording Society	Scheme data only	UK	1970–2015	242	255,354	202
Bryophytes	British Bryological Society *	Scheme and BRC data	UK	1970–2015	988	1,215,401	569
Caddisflies	Riverfly Recording Schemes: Trichoptera *	Scheme and BRC data	GB	1970–2014	186	164,102	123
Carabids	Ground Beetle Recording Scheme *	Scheme data only	UK	1970–2014	348	99,628	189
Centipedes	British Myriapod and Isopod Group, Centipede Recording Scheme	Scheme and BRC data	UK	1970–2015	51	23,315	22
Craneflies	Dipterists Forum, Cranefly Recording Scheme *	Scheme and BRC data	GB	1970–2015	344	80,620	157
Dragonflies	British Dragonfly Society, Dragonfly Recording Network *	Scheme data only	GB	1970–2015	54	734,573	41
Empid & Dolichopodid Flies	Dipterists Forum, Empididae, Hybotidae & Dolichopodidae Recording Scheme	Scheme data only	GB	1970–2015	649	92,049	228
Fungus Gnats	Dipterists Forum, Fungus Gnat Recording Scheme	Scheme and BRC data	UK	1970–2011	521	57,416	157
Gelechiid Moths	Gelechiid Recording Scheme	Scheme and BRC data	UK	1970–2013	152	75,972	86
Hoverflies	Dipterists Forum, Hoverfly Recording Scheme	Scheme data only	GB	1970–2014	272	537,873	219
Lacewings	Lacewings and Allies Recording Scheme *	Scheme, BRC and iRecord data	UK	1970–2015	82	20,713	30
Ladybirds	UK Ladybird Survey *	Scheme and BRC data	UK	1970–2015	47	128,184	37
Leaf and Seed Beetles	Chrysomelidae Recording Scheme *	Scheme, BRC and iRecord data	UK	1970–2015	271	67,051	145
Lichens	British Lichen Society *	Scheme data only	GB	1970–2015	2,142	877,741	700
Macro moths	National Moth Recording Scheme *	Scheme data only	UK	1970–2015	840	18,191,077	714
Mayflies	Riverfly Recording Schemes: Ephemeroptera *	Scheme and iRecord data	UK	1970–2015	45	13,9930	35
Millipedes	British Myriapod and Isopod Group, Millipede Recording Scheme *	Scheme and BRC data	UK	1970–2012	59	29,118	28
Non-marine Molluscs	Conchological Society of Great Britain and Ireland *	Scheme and BRC data	UK	1970–2015	257	154,108	129
Orthoptera	Grasshoppers and Related Insects Recording Scheme *	Scheme and BRC data	UK	1970–2015	28	65,269	24
Plant Bugs	Terrestrial Heteroptera Recording Scheme - Plant bugs and allied species *	Scheme, BRC and iRecord data	UK	1970–2015	413	129,594	222
Rove Beetles	Staphylinidae Recording Scheme	Scheme and BRC data	UK	1980–2015	796	27,757	79
Shield Bugs	Terrestrial Heteroptera Recording Scheme - Shield bugs and allied species *	Scheme, BRC and iRecord data	UK	1970–2015	66	27,011	39
Soldier Beetles	Soldier Beetles, Jewel Beetles and Glow-worms Recording Scheme *	Scheme, BRC and iRecord data	UK	1970–2015	58	21,943	31
Soldierflies	Soldierflies and Allies Recording Scheme *	Scheme data only	UK	1970–2015	148	48,301	83
Spiders	British Arachnological Society, Spider Recording Scheme	Scheme and BRC data	UK	1970–2014	626	441,767	402
Stoneflies	Riverfly Recording Schemes: Plecoptera *	Scheme and iRecord data	GB	1970–2015	32	56,613	25
Wasps	Bees, Wasps and Ants Recording Society	Scheme data only	UK	1970–2015	263	100,474	167
Weevils	Weevil and Bark Beetle Recording Scheme *	Scheme, BRC and iRecord data	UK	1970–2015	618	159,541	330
