# Supplementary material for: Annual estimates of occupancy for bryophytes, lichens and invertebrates in the UK, 1970–2015
Source: Sci Data. 2019 Nov 5;6:259. doi: 10.1038/s41597-019-0269-1 (PMC6831696; doi:10.1038/s41597-019-0269-1)

# Analysis Workflow

*Charlotte L. Outhwaite*

*16th May 2019*

## Contents

**Data Processing and Analysis Workflow** **1**
Data Collation . . . 1
Data Standardisation . . . 1
Organisation of Detection Histories . . . 6
The Occupancy Model . . . 7
Plotting Species Outputs . . . 9
Assessing Species Outputs . . . 9
The Posterior Distribution . . . 10
Species Trends . . . 11

## Data Processing and Analysis Workflow

This document describes the processes undertaken to produce the dataset of occupancy estimates for UK species outlined in the accompanying paper by Outhwaite et al. The document follows the structure of the methods section of the paper and more detail on each section can be found there.

### Data Collation

The raw occurrence records were collated from UK or GB recording schemes with additional data collected from the Biological Records Centre database and from the iRecord wildlife recording system. 29 schemes granted the use of their data which covered 31 broad groupings of species (see Table 1 of main document).

Some of this raw data is available through the NBN Atlas but this is scheme dependent. Since not all schemes have made their data publicly available we cannot share the raw datasets.

Each scheme organises its data in different ways so the initial scheme specific data processing cannot be included in this document. The scheme specific processing included the changes and groupings made to species names. These changes and the reasons for them are documented within the Species_Names.csv file within the repository. Changes or aggregations were necessary where there had been changes in taxonomy, differences in the names under which species were recorded or uncertainty in the identity of specific species.

This section resulted in the collation of 31 "raw" datasets, one for each major taxonomic grouping assessed.

### Data Standardisation

The "raw" datasets needed to be standardised so that all records fit the same criteria. These criteria were:

- Record location known to a 1km square grid cell.
- The date the record was taken was known.
- Records were from 1970 onward.
- Records were from the UK only.

The "raw" datasets were organised and columns specifically named so that the data standardisation process could be carried out using the same function across all datasets.

The data standardisation function and an example of its use on one of the 31 datasets are detailed here:

```r
# Standardising the raw data

# Load in the dataset.
# This has been reorganised from the raw scheme data to have consistent column names.
# Species name changes and aggregations have already taken place.
load("Ants_170203_processed_preclean.rdata")
# This is called taxa_data

# This is what the data looks like
str(taxa_data)
```

```
## 'data.frame':    56640 obs. of  4 variables:
##  $ CONCEPT     : Factor w/ 624 levels "","AGENIOIDEUS cinctellus",..: 290 290 292 292 341 341 435 43[illegible]
##  $ TO_GRIDREF  : Factor w/ 66723 levels "B782215","B8833",..: 22614 22614 33540 32722 13182 13609 13[illegible]
##  $ TO_STARTDATE: Factor w/ 20325 levels "0994-09-04","0998-01-01",..: 13906 14026 12890 12537 14251 [illegible]
##  $ TO_ENDDATE  : Factor w/ 20494 levels "0994-09-04","0998-12-31",..: 14064 14185 13043 12690 14665 [illegible]
```

```r
head(taxa_data)
```

```
##                             CONCEPT TO_GRIDREF TO_STARTDATE TO_ENDDATE
## 5847                   FORMICA rufa   SP277909   1993-07-25 1993-07-25
## 5848                   FORMICA rufa   SP277909   1994-03-20 1994-03-20
## 5849              FORMICA sanguinea   SU911590   1989-08-15 1989-08-15
## 5850              FORMICA sanguinea   SU855220   1988-04-23 1988-04-23
## 54455 Hypoponera punctatissima agg     SE2934   1995-01-01 1995-12-31
## 54456 Hypoponera punctatissima agg     SE5952   1925-01-01 1925-12-31
```

```r
# THE DATA STANDARDISATION FUNCTION

# Ensure all column names are as required
colnames(taxa_data) <- c("CONCEPT", "TO_GRIDREF", "TO_STARTDATE", "TO_ENDDATE")
```

`CONCEPT` identifies the species; `TO_GRIDREF` is the grid reference of the observation on the British or Irish national grid; `TO_STARTDATE` is the start date of the observation and `TO_ENDDATE` is the end date of the observation. Some observations might be assigned to a month or year, hence the start and end date columns. We only use records where the exact date is known (i.e. `TO_STARTDATE == TO_ENDDATE`).

```r
# Convert date columns to the correct date format.
# The original format of the date may vary by scheme
# so is outside of the standardisation function
taxa_data$TO_STARTDATE <- as.Date(taxa_data$TO_STARTDATE, format = "%Y-%m-%d")
taxa_data$TO_ENDDATE <- as.Date(taxa_data$TO_ENDDATE, format = "%Y-%m-%d")

# Load required libraries
library(sparta) # available on GitHub: https://github.com/biologicalrecordscentre/sparta
library(reshape2)
library(ggplot2)

# Data standardisation function
clean_data <- function(taxa,
                       site,
                       start_date,
                       end_date,
                       start_year,
                       end_year){
```

```r
# Organise the dataframe
taxa_data <- data.frame(taxa, site, start_date, end_date)

# Check that start_date == end_date
Before <- nrow(taxa_data)
taxa_data <- taxa_data[taxa_data$start_date == taxa_data$end_date,]
After <- nrow(taxa_data)
if(After < Before){
  warning(paste(Before-After,
                "rows have been removed because they don't have day precision"))
}


# Check that data has a precision of 1000 or less
taxa_data$precision <- sparta:::det_gr_precision(taxa_data$site)
Before <- nrow(taxa_data)
taxa_data <- taxa_data[taxa_data$precision <= 1000,]
After <- nrow(taxa_data)
if(After < Before){
  warning(paste(Before-After,
                "rows have been removed because they don't a precision of 1000 or less"))
}


# Reformat grid references to 1km2
# This function is within the sparta package
taxa_data$site <- sparta:::reformat_gr(taxa_data$site, prec_out = 1000)

# Remove precision column so that duplicates are not masked after grid ref reformat
taxa_data <- taxa_data[, c(1:4)]

# Remove any duplicates that are now in the data from changing grid ref precision
Before <- nrow(taxa_data)
taxa_data <- unique(taxa_data)
After <- nrow(taxa_data)
if(After<Before){
  warning(paste(Before-After,
                "rows have been removed as duplicates"))
}

# Only select time frame of interest - eg. 1970-2015
# Adds a column for just the year from one of the date columns
taxa_data$YEAR <-as.numeric(format(taxa_data$start_date, "%Y"))

Before <- nrow(taxa_data)
taxa_data <- taxa_data[taxa_data$YEAR >= start_year & taxa_data$YEAR <= end_year,]
After <- nrow(taxa_data)
if(After<Before){
  warning(paste(Before-After,
                "rows have been removed as not within desired time period",
                start_year, "-", end_year))
}
```

```r
  # Checking that only UK grid cells are included.

  # Read in table detailing grid cell country.
  UK_cells <- read.csv("sq1km_country_id_UKonly_bordercells_tosmallercountry.csv")

  Before <- nrow(taxa_data)
  taxa_data <- taxa_data[taxa_data$site %in% UK_cells$SQ1_SQUARE,]
  After <- nrow(taxa_data)
  if(After < Before){
    warning(paste(Before-After,
                  "rows have been removed because the grid cells are not in the UK"))
  }

  # Standardise the column names
  colnames(taxa_data) <- sub('taxa', 'CONCEPT', colnames(taxa_data))
  colnames(taxa_data) <- sub('end_date', 'TO_ENDDATE', colnames(taxa_data))
  colnames(taxa_data) <- sub('start_date', 'TO_STARTDATE', colnames(taxa_data))
  colnames(taxa_data) <- sub('site', 'TO_GRIDREF', colnames(taxa_data))

  # Refactor the concept column
  taxa_data$CONCEPT <- factor(taxa_data$CONCEPT)

  # Return data
  return(taxa_data)

} # End of data standardisation function
```

We can use this function to standardise each dataset.

```r
# Run the function across the dataset
taxa_data <- clean_data(taxa = taxa_data$CONCEPT ,
                        site = taxa_data$TO_GRIDREF,
                        start_date = taxa_data$TO_STARTDATE,
                        end_date = taxa_data$TO_ENDDATE,
                        start_year = 1970,
                        end_year = 2015)


# View the output
str(taxa_data)
```

```
## 'data.frame':    34597 obs. of  5 variables:
##  $ CONCEPT     : Factor w/ 60 levels "FORMICA aquilonia",..: 9 9 11 11 35 35 35 35 35 37 ...
##  $ TO_GRIDREF  : chr  "SP2790" "SP2790" "SU9159" "SU8522" ...
##  $ TO_STARTDATE: Date, format: "1993-07-25" "1994-03-20" ...
##  $ TO_ENDDATE  : Date, format: "1993-07-25" "1994-03-20" ...
##  $ YEAR        : num  1993 1994 1989 1988 1987 ...
```

```r
head(taxa_data)
```

```
##               CONCEPT TO_GRIDREF TO_STARTDATE TO_ENDDATE YEAR
## 1        FORMICA rufa     SP2790   1993-07-25 1993-07-25 1993
## 2        FORMICA rufa     SP2790   1994-03-20 1994-03-20 1994
## 3   FORMICA sanguinea     SU9159   1989-08-15 1989-08-15 1989
## 4   FORMICA sanguinea     SU8522   1988-04-23 1988-04-23 1988
```

```
## 10 MYRMICA lobicornis     SD7666   1987-05-30 1987-05-30 1987
## 11 MYRMICA lobicornis     SK4691   1982-07-20 1982-07-20 1982
```

The data standardisation process resulted in 31 "standardised" datasets where records were of the same precision. Table 1 in the main document details information on these "standardised datasets".

Plotting the location of these records shows the country coverage of the data. These maps have been supplied for each of the 31 groups in the supplementary information.

```
# Plotting the spatial spread of the dataset

# Take the grid cells
gridrefs <- taxa_data$TO_GRIDREF

# Reformat grid refs to 10km level so that they are more visible when plotted
gridrefs <- as.data.frame(sparta:::reformat_gr(gridrefs, 10000))

# Edit column name
colnames(gridrefs) <- "ref"

# Calculate the densities at each 10km cell

# Space to save densities
all_res <- NULL

for(gr in unique(gridrefs$ref)){

  sub <- gridrefs[gridrefs$ref == gr, ]
  total <- length(sub)

  result <- c(gr, total)

  all_res <- rbind(all_res, result)
}

# Convert to dataframe
all_res <- as.data.frame(all_res)
colnames(all_res) <- c("gridref", "total")

all_res$total <- as.numeric(as.character(all_res$total))


# Load UK data
data(UK)

# Plot UK outline
sparta:::plot_GIS(sparta:::UK,
                  new.window = FALSE,
                  show.grid = FALSE,
                  show.axis = FALSE,
                  xlab = "",
                  ylab = "",
                  main = "Ants",
                  cex.main = 1)
```

```r
# Add these grid references to the exisiting plot
sparta:::plotUK_gr(all_res$gridref, col="red", border = "blue", density = all_res$total)
```

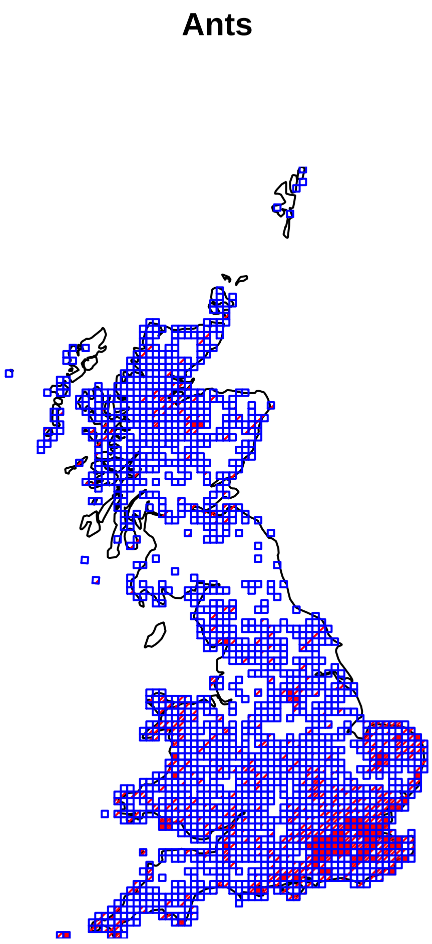

## Organisation of Detection Histories

In order to fit the occupancy model, the "standardised" data needed to be arranged into detection histories. This represents a visit by species matrix populated with 0s and 1s to denote non-detections and detections of the species at each site/year combination included in the dataset.

The function to carry out this process is available in the R package sparta. This package is available for download from Github (https://github.com/BiologicalRecordsCentre/sparta). This function also calculates

the list length information required within the model. List length is the number of species recorded during a particular visit (unique site/year combination).

```
# Organise the data into detection histories

# Use the formatOccData function from the sparta package to create the detection histories
visitData <- formatOccData(taxa = taxa_data$CONCEPT,
                           site = taxa_data$TO_GRIDREF,
                           survey = taxa_data$TO_STARTDATE)

# Take a look at the two datasets producted from the function.

# 1. Detection history, taken first 4 columns only,
# there are columns appended for each species
head(visitData$spp_vis[, 1:4])
```

```
##              visit FORMICA aquilonia FORMICA cunicularia FORMICA exsecta
## 1  D08432007-10-10             FALSE               FALSE           FALSE
## 2  D29112006-09-16             FALSE               FALSE           FALSE
## 3 HP60132004-07-13             FALSE               FALSE           FALSE
## 4 HP60132006-07-18             FALSE               FALSE           FALSE
## 5 HU23502006-07-20             FALSE               FALSE           FALSE
## 6 HU46412004-07-13             FALSE               FALSE           FALSE
```

This table details which species were observed on each visit. `visit` is a unique combination of 1km square (the grid reference) and date. Subsequent columns detail, for each species, whether it was observed or not.

```
# 2. List length information
head(visitData$occDetdata)
```

```
##              visit   site L   TP
## 1  D08432007-10-10  D0843 1 2007
## 2  D29112006-09-16  D2911 1 2006
## 3 HP60132004-07-13 HP6013 1 2004
## 4 HP60132006-07-18 HP6013 1 2006
## 5 HU23502006-07-20 HU2350 1 2006
## 6 HU46412004-07-13 HU4641 1 2004
```

This table details the metadata of each visit. `visit` is a unique combination of 1km square (the grid reference) and date. `site` is the 1km grid reference. `L` is the list length for the visit, i.e. the number of species observed on that visit. `TP` is the 'time period' in which the observation was made, in this case the year of the observation.

## The Occupancy Model

The detection histories and list length information is then fed into the occupancy model. Again, this process can be carried out using the R package sparta, using the function `occDetFunc`.

Details of the occupancy model can be found in the manuscript. Information on additional options for the occupancy model can be found in the sparta help files. The model upon which this is based was tested in a paper by Outhwaite et al (2018), Prior specification in Bayesian occupancy modelling improves analysis of species occurrence data, Ecological Indicators.

Here, we run the occupancy model for the UK, including estimating parameters for a number of sub regions. This file assigns 1km squares to a country. Where a square crosses a border it is assigned to the smaller country.

```
# This is a simple table detailing which grid cells are in which country.
# The table is filled with 1s and NAs.
```

```r
regional_codes <- read.csv("sq1km_country_id_GBonly_bordercells_tosmallercountry.csv")

head(regional_codes)
```

```
##   SQ1_SQUARE ENGLAND WALES SCOTLAND
## 1     HP4700      NA    NA        1
## 2     HP4701      NA    NA        1
## 3     HP4702      NA    NA        1
## 4     HP4703      NA    NA        1
## 5     HP4704      NA    NA        1
## 6     HP4705      NA    NA        1
```

We use the function `occDetFunc` to run the occupancy model:

```r
# Running the occupancy model

# Take a look at regional_codes
head(regional_codes)
```

```
##   SQ1_SQUARE ENGLAND WALES SCOTLAND
## 1     HP4700      NA    NA        1
## 2     HP4701      NA    NA        1
## 3     HP4702      NA    NA        1
## 4     HP4703      NA    NA        1
## 5     HP4704      NA    NA        1
## 6     HP4705      NA    NA        1
```

```r
# Run the occupancy model for one species of Ant using the following code
# NOTE: this takes a few hours to run
out <- occDetFunc(taxa_name = "FORMICA rufa",
                  occDetdata = visitData$occDetdata,
                  spp_vis = visitData$spp_vis,
                  n_iterations = 40000,
                  nyr = 2,
                  burnin = 20000,
                  thinning = 3,
                  n_chains = 3,
                  write_results = TRUE,
                  regional_codes = regional_codes,
                  region_aggs = list(GB = c('ENGLAND', 'WALES', 'SCOTLAND')),
                  modeltype = c("ranwalk", "halfcauchy", "catlistlength"),
                  return_data = TRUE)
```

```r
# View output summary, note that this includes all model variables.
head(out$BUGSoutput$summary)
```

```
##                    mean       sd      2.5%       25%       50%       75%
## a_ENGLAND[1] -11.23780 2.399811 -16.31207 -12.83232 -11.01671 -9.479368
## a_ENGLAND[2] -11.21184 2.294548 -16.04442 -12.77074 -11.00142 -9.496505
## a_ENGLAND[3] -11.29264 2.243598 -16.01261 -12.84111 -11.09757 -9.627947
## a_ENGLAND[4] -11.31409 2.201021 -15.92704 -12.85304 -11.09885 -9.631987
## a_ENGLAND[5] -11.23453 2.138984 -15.66508 -12.76378 -11.00102 -9.549906
## a_ENGLAND[6] -11.07891 2.100933 -15.37270 -12.60469 -10.85106 -9.403491
##                   97.5%     Rhat n.eff
## a_ENGLAND[1] -7.122105 1.324412    10
## a_ENGLAND[2] -7.287927 1.361423     9
```

```
## a_ENGLAND[3] -7.488927 1.387131     9
## a_ENGLAND[4] -7.668891 1.412612     9
## a_ENGLAND[5] -7.773850 1.443885     8
## a_ENGLAND[6] -7.711988 1.455953     8
```

### Plotting Species Outputs

Using the `plot` function in `sparta` on the output from the `occDetFunc` function will produce a plot of the mean occupancy and associated 95% credible intervals for that species. The function will colour the points according to their rhat value.

```
# Using the plot function in sparta
plot(out)
```

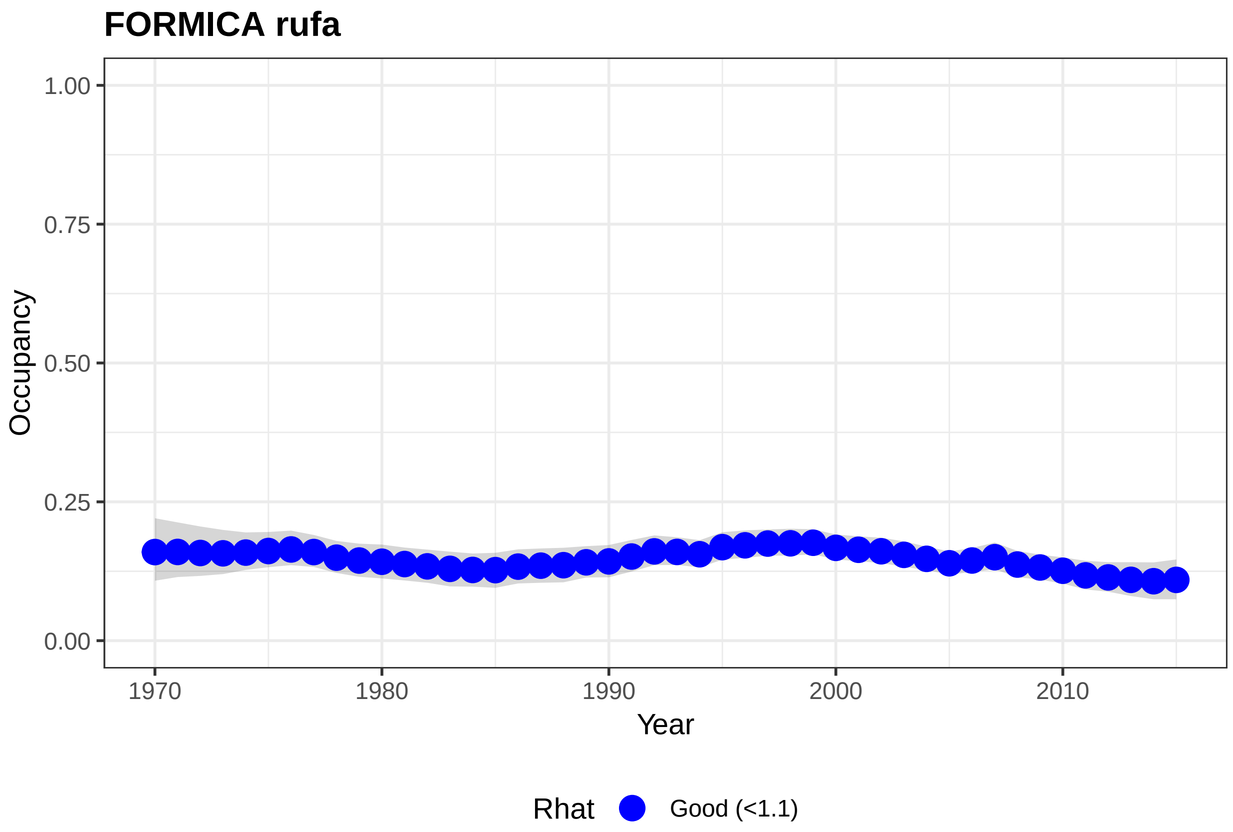


### Assessing Species Outputs

Species were assessed on their number of records. If a species had fewer than 50 records contributing to their output then they were not considered to contain valuable information on trends. Species were also dropped if they have a gap of no records for 10 or more years.

```
# Number of observations
out$species_observations
```

```
## [1] 965
```

```
# In how many years is each site visited?
yps <- rowSums(acast(visitData$occDetdata, site ~ TP, length, value.var = "L") > 0)
```

```
# Sites must have records from at least 2 years
nyr = 2
sites_to_include <- names(yps[yps >= nyr])

# Subset to those sites
taxa_data <- taxa_data[taxa_data$TO_GRIDREF %in% sites_to_include, ]

# Get the records for the species of interest
sp_recs <- taxa_data[taxa_data$CONCEPT == "FORMICA rufa", ]

# Which years does the species have records for?
years_with_data <- sort(unique(sp_recs$YEAR))

# What are the gaps between these?
gaps_between_years <- diff(years_with_data)

# Is the biggest gap 10 or more years?
max(gaps_between_years) >= 10
```

```
## [1] FALSE
```

```
# In this case the biggest gap is less than 10 years
max(gaps_between_years)
```

```
## [1] 2
```

## The Posterior Distribution

Since the posterior distributions for the model outputs are so large, we have chosen to use and supply 1000 samples from the complete distribution. This makes analyses much more manageable.

```
# Select 1000 samples from the posterior distribution

# This selects the posterior for the occupancy estimates (psi.fs) only
raw_occ <- data.frame(out$BUGSoutput$sims.list)

# Get the occupancy estimates from the summary table
raw_occ <- raw_occ[, grep('psi.fs.r', colnames(raw_occ))]
post <- raw_occ[sample(1:nrow(raw_occ), 1000),]

# Add metadata to our data
post$spp <- "FORMICA rufa"
post$iter <- 1:1000

# Take a look at the posterior samples
# Here just the first 5 columns are shown.
# There is a column for each region/year combination.
head(post)[1:5]
```

```
##       psi.fs.r_ENGLAND.1 psi.fs.r_ENGLAND.2 psi.fs.r_ENGLAND.3
## 12595          0.1550725          0.1454106          0.1637681
## 17363          0.1570048          0.1550725          0.1671498
## 5298           0.1265700          0.1599034          0.1850242
## 5279           0.1628019          0.1632850          0.1758454
## 8224           0.1463768          0.1570048          0.1719807
## 8763           0.2492754          0.2502415          0.2231884
```

```
##       psi.fs.r_ENGLAND.4 psi.fs.r_ENGLAND.5
## 12595          0.1657005          0.1608696
## 17363          0.1661836          0.1806763
## 5298           0.1560386          0.1903382
## 5279           0.1855072          0.1782609
## 8224           0.1521739          0.1710145
## 8763           0.2009662          0.1922705
```

```
ncol(post)
```

```
## [1] 186
```

The column names give the details of the occupancy estimates across a random sample of 1000 iterations after thinning. The prefix `psi.fs.r_` defines these columns as occupancy estimates for regions. This is followed by the region (i.e. `ENGLAND`), and the year (i.e. `.1`), where `1` is the first year in the dataset (saved as `out$min_year` for reference).

## Species Trends

Species trends were estimated as the annual percentage growth rate using the first and last years for which that species had records. For national estimates of growth rate, the posterior samples for the occupancy estimate at the highest level (either GB or UK depending on dataset coverage) were used.

```
# Estimate species trends

# Need to determine which years are the first and last with records of
# the species of interest.

# First, process records data so that sites with less than nyr visits are removed.
# This is usually done within the occDetFunc function.

# Number of visits a site must have to be included.
nyr = 2

# Name of the example species
species <- "FORMICA rufa"

# Extract visit data elements created from formatOccDet function above
taxa_name <- as.character(species)
occDetdata = visitData$occDetdata
spp_vis = visitData$spp_vis

# Add the focal column (was the species recorded on the visit?).
# Use the spp_vis dataframe to extract this info.
occDetdata <- merge(occDetdata, spp_vis[,c("visit", taxa_name)])
names(occDetdata)[names(occDetdata) == taxa_name] <- "focal"

# Only include sites which have more than nyr of records
yps <- rowSums(acast(occDetdata, site ~ TP, length, value.var = 'L') > 0)
sites_to_include <- names(yps[yps >= nyr])
i <- occDetdata$site %in% sites_to_include

# Subset the records to only those sites with sufficient visits
subset_recs <- occDetdata[i, ]

# Subset again to those where the species of interest was recorded
```

```r
subset_recs <- subset_recs[subset_recs$focal == TRUE, ]

subset_recs$species <- species

# What is our year range of interest.
years <- 1970:2015

# What years are represented within the data
data_years <- table(subset_recs$TP)

# Number of years with no data
missing_yrs <- length(years) - sum(years %in% names(data_years))

# First year with data
first_yr <- min(as.numeric(names(data_years)))

# Last year with data
last_yr <- max(as.numeric(names(data_years)))

# Function for annual growth rate
annual_growth_rate <- function(first, last, nyrs){

  (((last/first)^(1/nyrs))-1)*100

}

# Extract the posterior samples for the GB estimates only.
# This is the highest level that estimates are generated for from the ant dataset.
sp_post <- post[, grep("GB", colnames(post))]

colnames(sp_post) <- 1970:2015

# Subset to only those years with records
sp_post <- sp_post[, as.character(first_yr:last_yr)]

# Select the relevant columns from the posterior to estimate the growth rate
first <- sp_post[, 1]
last <- sp_post[, ncol(sp_post)]
nyr <- ncol(sp_post)

# Calculate annual growth rate
# This will calculate 1000 estimates of the growth rates, from each set of iterations
rates <- annual_growth_rate(first, last, nyr)

# Estimate the mean to 4 decimal places
ann_growth_rate <- round(mean(rates), 4)

# Estimate the credible intervals from the posterior samples
CI_lower <- quantile(rates, probs = 0.025, na.rm = TRUE)
CI_upper <- quantile(rates, probs = 0.975, na.rm = TRUE)

# Present the results
results <- cbind(ann_growth_rate, CI_lower, CI_upper)
```

```r
rownames(results) <- NULL
results
```

```
##      ann_growth_rate  CI_lower  CI_upper
## [1,]         -0.7968 -1.898997 0.1933711
```

A box plot of the results shows the range of estimates more clearly:

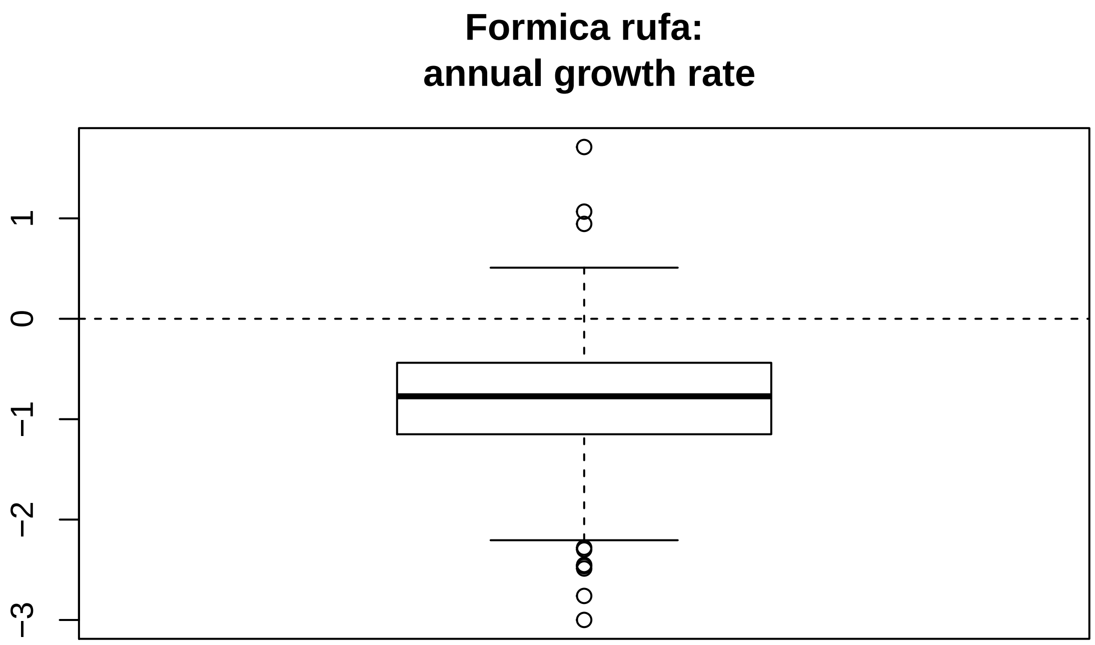

Supplement: Supplementary file 1 [file 41597_2019_269_MOESM1_ESM.pdf]
