# Supplementary material for: Annual estimates of occupancy for bryophytes, lichens and invertebrates in the UK, 1970–2015
Source: Sci Data. 2019 Nov 5;6:259. doi: 10.1038/s41597-019-0269-1 (PMC6831696; doi:10.1038/s41597-019-0269-1)

Supplementary Figure S1:
Maps of the coverage of records in each input dataset,
blue grid cells show the 10km square that includes 1km square records,
[illegible]e red shading represents the number of records within that 10 km square.
The more densely shaded areas indicate areas with high levels of recording.

Ants

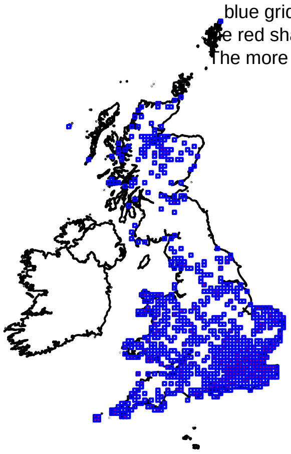

AquaticBugs

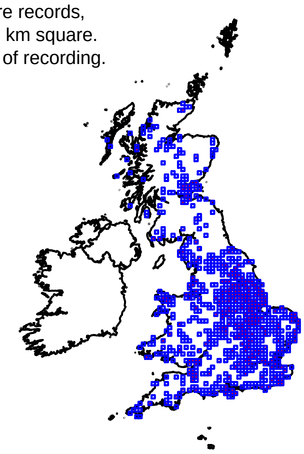

Bees

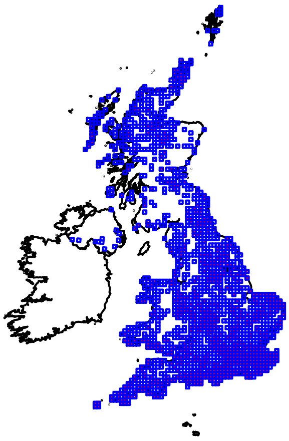

Bryophytes

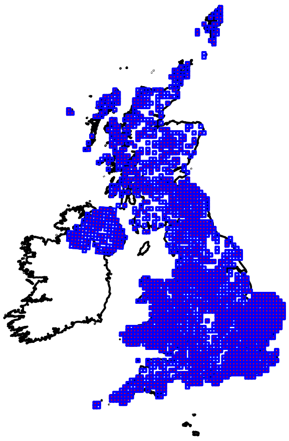

Carabids

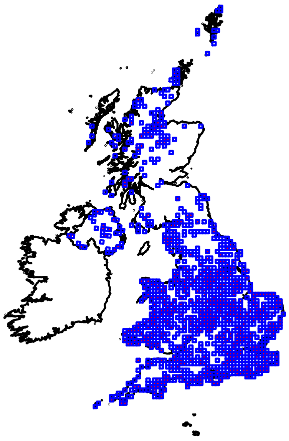

Centipedes

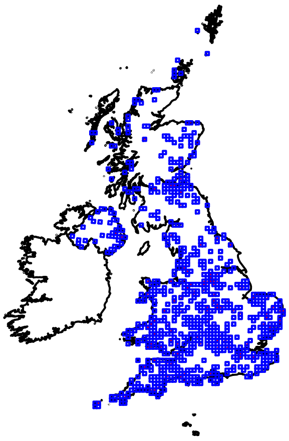

Craneflies

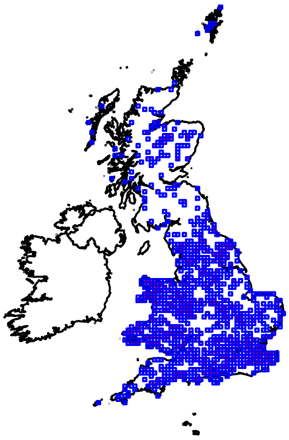

Dragonflies

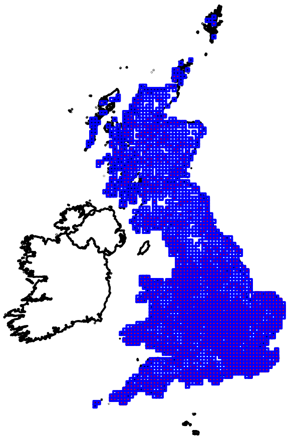

Empid&DolichopodidFlies

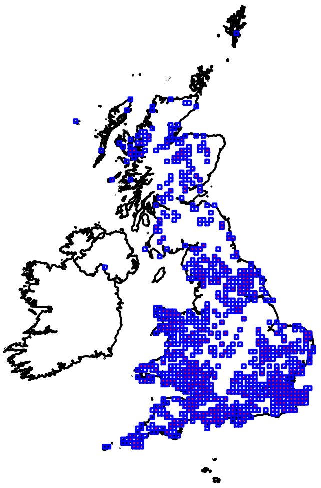

Mayflies

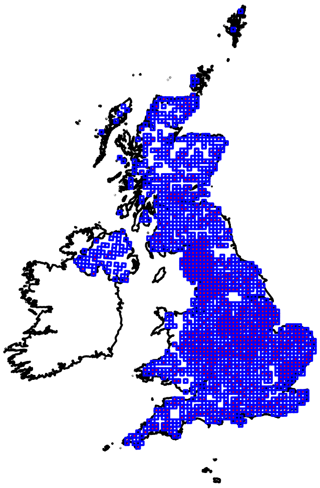

FungusGnats

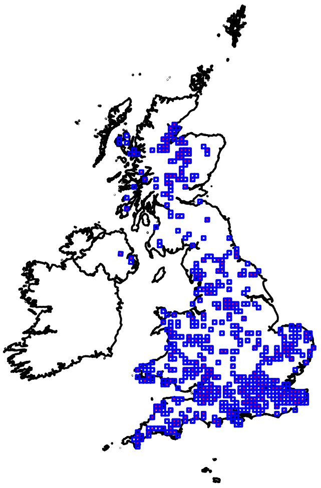

Gelechiids

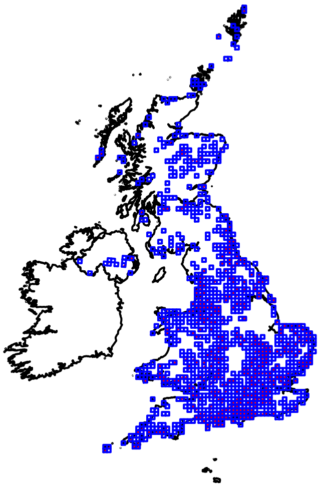

Hoverflies

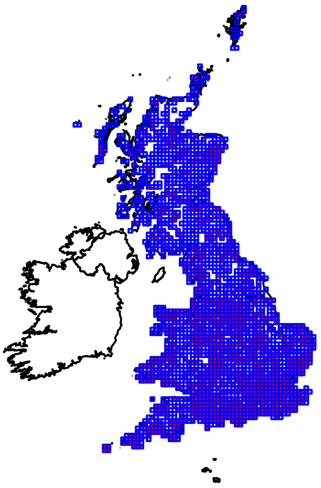

Ladybirds

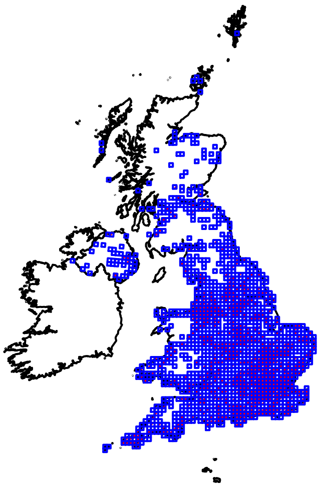

Leaf&SeedBeetles

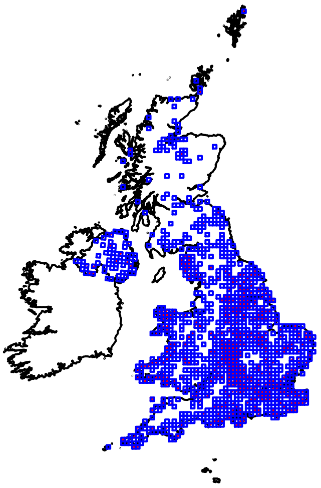

Lichens

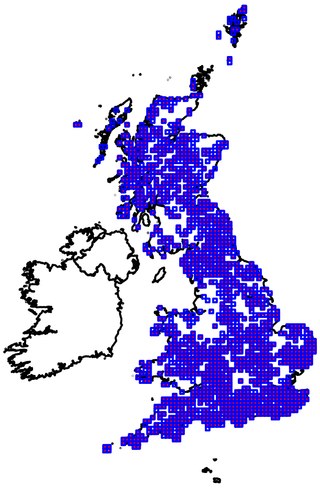

Millipedes

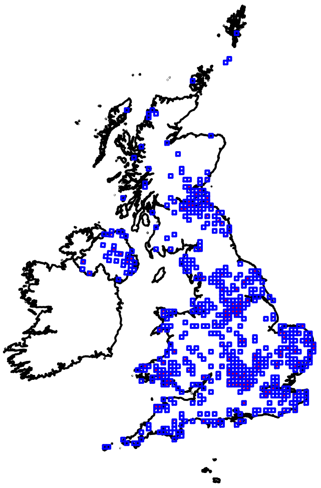

NonmarineMolluscs

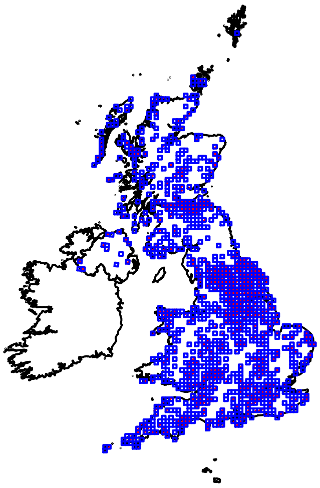

Moths

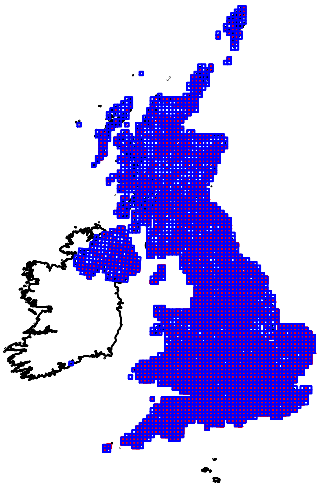

Lacewings

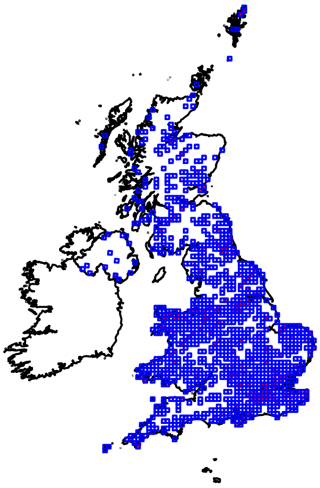

Orthoptera

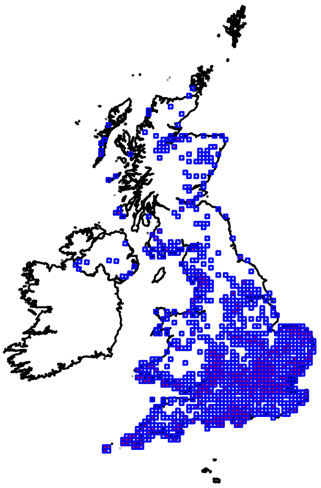

PlantBugs

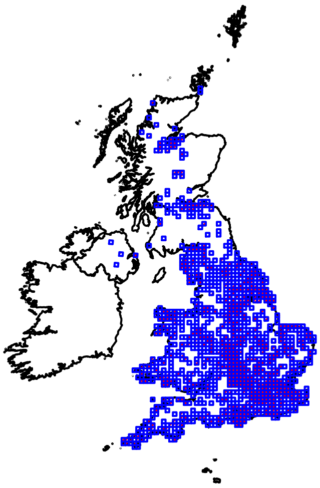

Stoneflies

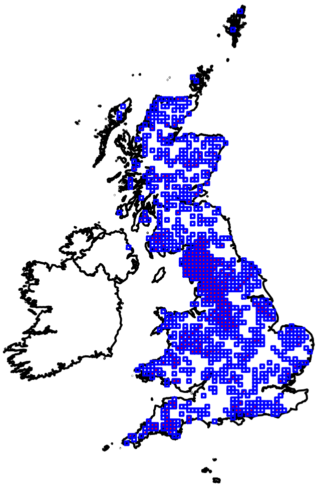

RoveBeetles

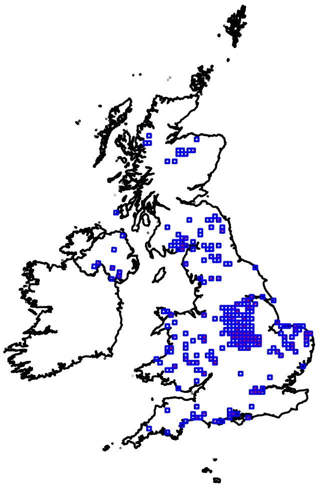

ShieldBugs

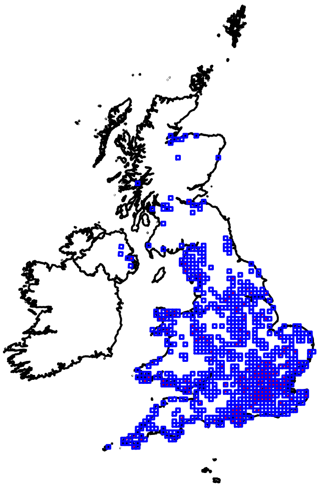

SoldierBeetles

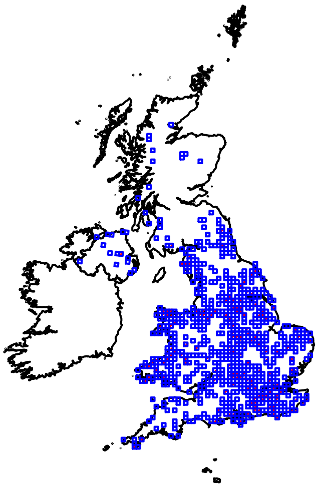

Soldierflies

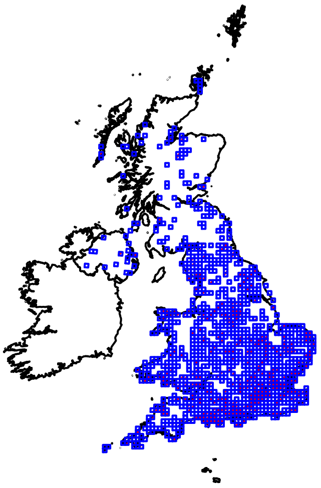

Spiders

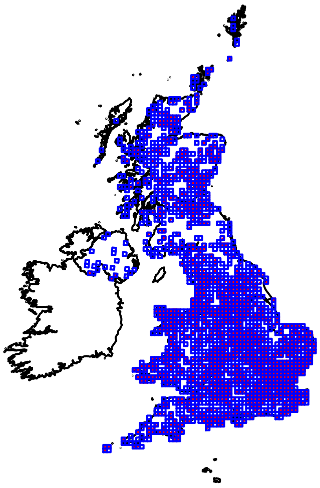

Caddisflies

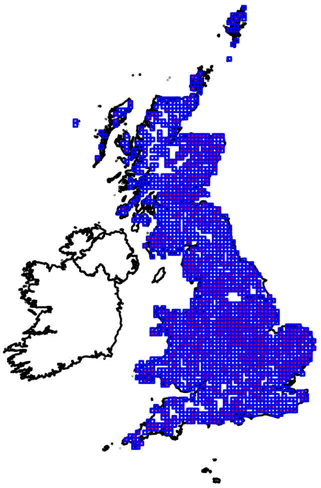

Wasps

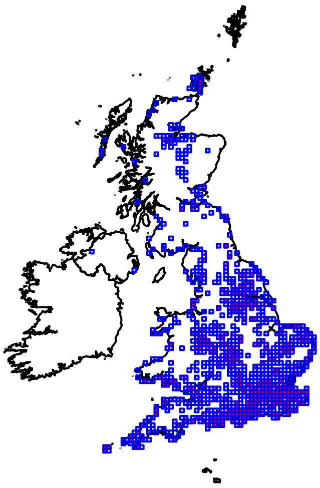

Weevils

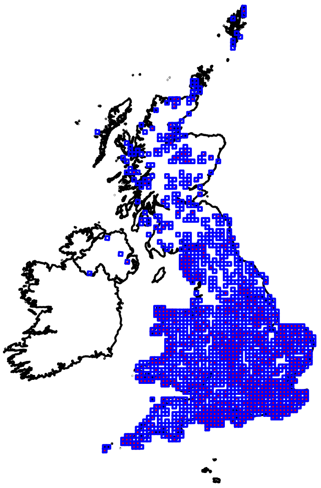

Supplement: Supplementary file 2 — Supplementary Figure S1. [file 41597_2019_269_MOESM2_ESM.pdf]
